# Surgical management and clinical reflections on acute type A intramural hematoma with a focal intimal tear in an adult with double aortic arch: a case report

**DOI:** 10.1186/s12893-026-03680-y

**Published:** 2026-06-30

**Authors:** Dequan Zhu, Jun Zhou, Chuanzhen Wan, Xiaofei Ren, Wei Wang

**Affiliations:** 1 Department of Cardiothoracic and Vascular Surgery, Taihe Hospital (Hubei University of Medicine), Shiyan, Hubei 442000 China; 2 Department of Clinical Medicine, Shandong Second Medical University, Weifang, 261053 Shandong China

**Keywords:** Double aortic arch, Acute aortic syndrome, Type A intramural hematoma, Vascular ring, Surgical management

## Abstract

**Background:**

Double aortic arch (DAA) is a rare congenital aortic arch anomaly that is usually identified in infancy because of symptoms related to a vascular ring. Acute type A intramural hematoma (IMH) with a focal intimal tear in an adult with DAA is extremely rare, and no consensus has been established regarding emergency management.

**Case presentation:**

Seventy-three year-old woman presented with burning pain in the throat and suprasternal notch and was initially suspected of having acute coronary syndrome. Computed tomography angiography (CTA) of the whole aorta and supra-aortic vessels demonstrated a double aortic arch, with the right common carotid artery and right subclavian artery originating from the right arch and the left common carotid artery and left subclavian artery originating from the left arch. After preoperative evaluation, emergency surgery was performed through a median sternotomy. Intraoperatively, a focal intimal tear approximately 2 cm in length was identified in the ascending aorta. Given the complex branching anatomy of the double aortic arch, right femoral artery cannulation was used to establish cardiopulmonary bypass in order to minimize invasive manipulation of the arch. Resection of the diseased ascending aorta and graft replacement were performed, and the potential false lumen at the aortic root was obliterated using the adventitial inversion technique. The prosthetic graft was wrapped with bovine pericardium, and the double aortic arch was not addressed during the same operation. The patient experienced recurrent perioperative hypoxemia and was extubated 17 h after surgery following respiratory support, lung-protective management, and anti-inflammatory treatment. She was discharged on postoperative day 12. Approximately 1 month later, she was readmitted with chest pain, and CTA revealed a newly developed dissection in the proximal right aortic arch. The family declined reoperation, and the patient was subsequently lost to follow-up.

**Conclusions:**

In patients with DAA complicated by acute type A IMH with a focal intimal tear, limited ascending aortic replacement in the emergency setting may reduce surgical trauma; however, it may leave a high-risk residual arch segment and increase the risk of clamp-related injury or insufficient resection margins. Perioperative airway compression caused by the vascular ring should be assessed using imaging, and one-stage or staged reconstruction should be planned according to the patient's condition. Strict postoperative blood pressure control and close follow-up are essential to reduce the risk of recurrence.

## Background

Acute aortic syndrome (AAS) refers to a group of life-threatening aortic emergencies that mainly include classic aortic dissection (AD), intramural hematoma (IMH), and penetrating atherosclerotic ulcer (PAU). Its incidence is approximately 7.7 per 100,000 person-years, and IMH accounts for about 4%–22% of all AAS cases [1, 2]. Type A IMH is associated with a high risk of rupture, cardiac tamponade, and other catastrophic complications, and generally requires urgent surgical repair [3, 4].

Double aortic arch is caused by abnormal regression of the fourth pharyngeal arch arteries and accounts for less than 1% of congenital heart disease. It is the most common form of complete vascular ring. Most cases are detected in infancy because of symptoms related to tracheal or esophageal compression, such as stridor, croup-like cough, recurrent respiratory tract infection, respiratory distress, and dysphagia. Among these children, approximately 12%–50% also have other congenital cardiovascular anomalies [5, 6]; in adult cohort studies, the detection rate of DAA is only 0.44% [7]. Reports of DAA complicated by AAS are scarce and have mostly involved type B dissection or descending aortic disease. Adult DAA complicated by proximal aortic disease is particularly rare.

Here, we report a 73-year-old woman with double aortic arch complicated by acute type A IMH with a focal intimal tear. From the perspective of surgical decision-making, and in the context of advanced age, complex arch anatomy, the paucity of similar cases, and current guideline recommendations, we discuss and reflect on the surgical strategy and postoperative complications in patients with DAA complicated by type A AAS.

## Case presentation

**Fig. 1:**
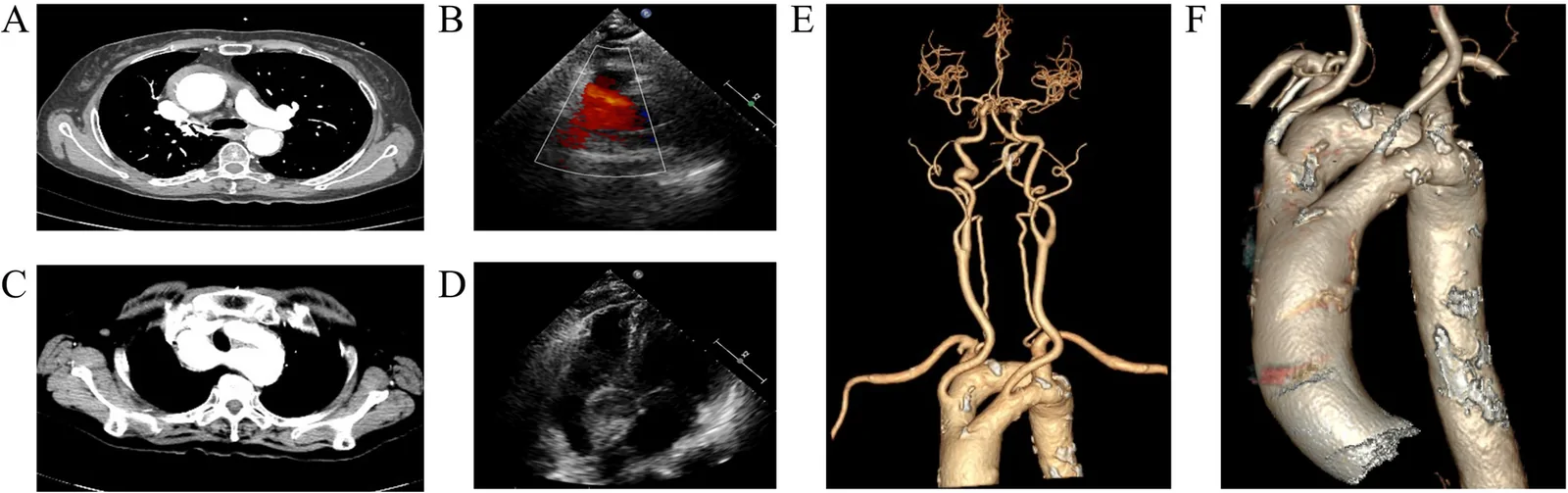
**A**, **C** Emergency CTA demonstrates a double aortic arch; an ulcer-like lesion in the ascending aorta suggests possible PAU. **B**, **D** Echocardiography shows no concomitant congenital cardiac malformation. **E**, **F** Reconstructed images highlight the complex supra-aortic branch anatomy

A 73-year-old woman had a long-standing history of hypertension and diabetes mellitus. She had been taking antihypertensive and hypoglycemic medications regularly for many years and reported that her blood pressure had been well controlled. She presented with a burning pain in the throat and suprasternal notch that had persisted for 12 h and worsened over the previous 2 h. Electrocardiography showed atrial fibrillation and abnormal Q waves in leads III and aVF. Blood pressure in the left upper limb was 149/83 mmHg. Given her history of diabetes, acute coronary syndrome was initially suspected. Physical examination revealed no obvious chest tenderness or pathological cardiac murmurs. Non-contrast chest computed tomography suggested an acute aortic syndrome and incidentally revealed a double aortic arch. Emergency whole-aorta CTA showed a crescent-shaped low-density area along the lateral wall of the ascending aorta, with a maximal diameter of approximately 5.0 cm. The hematoma extended from the aortic root to the aortic arch. The right common carotid artery and right subclavian artery originated from the right aortic arch, whereas the left common carotid artery and left subclavian artery originated from the left aortic arch. The right arch was dominant and the left arch was smaller, with no common trunk. No dissection was identified within the double aortic arch (Fig. 1). The patient was then transferred to the intensive care unit (ICU).

During ICU observation, the patient experienced recurrent hypoxemia. Arterial blood gas analysis showed a pH of 7.419, a PaCO₂ of 32.6 mmHg, and a PaO₂ of 51.6 mmHg, with slight improvement after high-flow nasal oxygen therapy. Bedside echocardiography revealed no cardiac malformation or valvular insufficiency (Fig. 1). To clarify the cerebral perfusion pattern and plan the surgical strategy, CTA of the head and neck was performed and showed that the intramural hematoma involved the proximal-to-mid portions of both common carotid arteries and the proximal right subclavian artery. A double aortic arch was present, no brachiocephalic trunk was identified, and the proximal segments of both common carotid arteries and subclavian arteries were tortuous, stiff-walled, and calcified (Fig. 1).

**Fig. 2:**
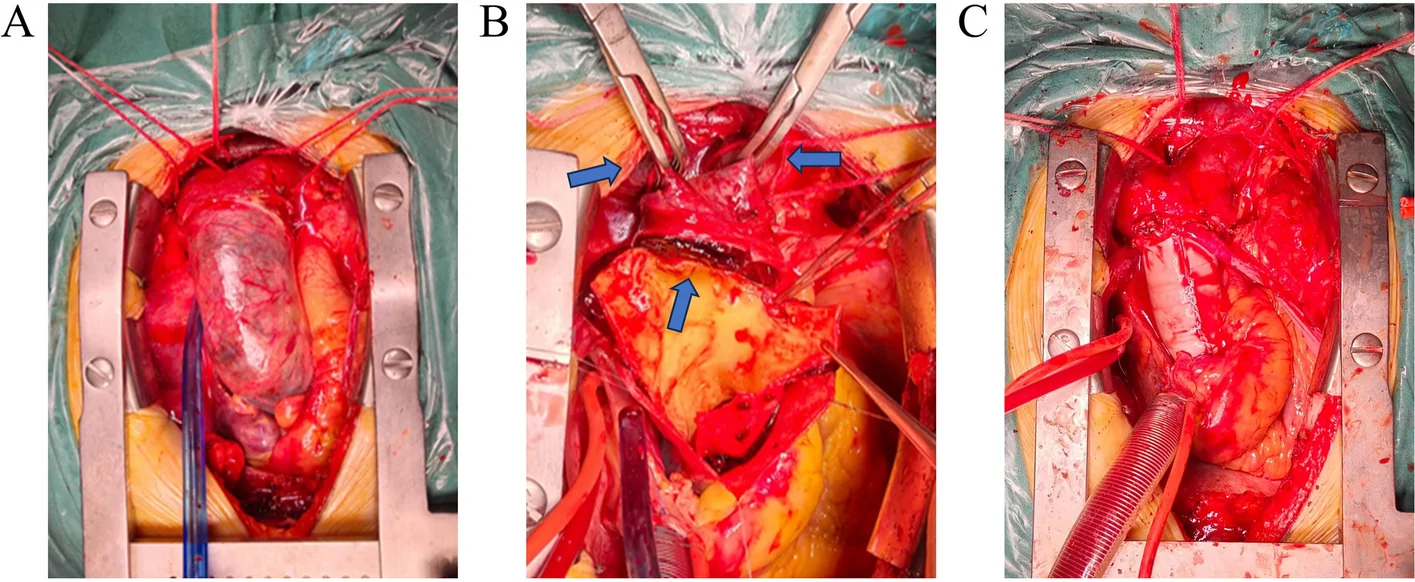
**A** Intraoperative view showing double aortic arch malformation and periaortic intramural hematoma. **B** Focal intimal defect in the ascending aorta. **C** Prosthetic graft wrapped with bovine pericardium

After completion of the preoperative preparation, emergency surgery was performed under general anesthesia through a median sternotomy. Intraoperative exploration showed aneurysmal dilatation of the ascending aorta, a thinned aortic wall, and extensive peri-aortic hematoma. The aortic arch was exposed and the double aortic arch anomaly was confirmed (Fig. 2). The right femoral artery was cannulated. Bicaval venous drainage was established through a right atrial appendage incision, and a left ventricular vent was inserted through the right superior pulmonary vein. Cardiopulmonary bypass was then established. After adequate left ventricular decompression, both arches were clamped simultaneously at their origins. Upon opening the ascending aorta, intramural hematoma was identified without a typical intimal flap. The aortic valve and coronary ostia were not involved. A focal intimal defect approximately 2 cm in length was found in the distal ascending aorta (Fig. 2); based on the preoperative CTA findings, concomitant penetrating atherosclerotic ulcer was considered possible. After resection of the diseased ascending aortic segment and evacuation of the intramural hematoma, the aortic root was managed first: the aorta was transected 1.5 cm above the coronary ostia, and the aortic root wall was reinforced using the adventitial inversion technique to obliterate the potential interlaminar false lumen. The ascending aorta was then transected 1 cm proximal to the origins of both arches. A straight vascular graft was completely wrapped with bovine pericardium (Fig. 2). Distal end-to-end anastomosis to the ascending aorta was performed with continuous 4–0 Prolene sutures, followed by proximal anastomosis of the graft to the aortic root. After completion, the clamps were released and circulation was resumed; following de-airing, spontaneous cardiac rhythm returned. No intraluminal arch exploration was performed, and the clamped segments were not included in the resection. Finally, biological sealant was sprayed over the surgical field to reduce oozing. At the end of the procedure, one drain was placed in the anterior mediastinum and one in the pericardial cavity, with no pleural chest tubes inserted. Cerebral oxygen saturation and cerebral perfusion were monitored throughout the operation using near-infrared spectroscopy and transcranial Doppler, and no significant drop in cerebral oxygen saturation was observed. Deep hypothermic circulatory arrest was not used. Cardiopulmonary bypass (CPB) time was 113 min and aortic cross-clamp time was 70 min. Autologous blood salvage amounted to 320 mL, and no allogeneic blood transfusion was administered. The double aortic arch was not resected and the vascular ring was not divided; only the necessary dissection and clamping maneuvers were performed.

Postoperatively, the patient returned to the ICU and received continued mechanical ventilation (SIMV, FiO2 45%), together with a short course of corticosteroid pulse therapy, inhaled nitric oxide, and intravenous sivelestat sodium to improve oxygenation and reduce the inflammatory response. After comprehensive assessment, the patient met extubation criteria and was extubated 17 h after surgery. After extubation, the patient received high-flow nasal oxygen therapy (flow rate, 60 L/min; FiO2, 60%), followed by conventional nasal cannula oxygen. Arterial blood gas analysis after extubation showed a pH of 7.447, a PaCO₂ of 28.3 mmHg, and a PaO₂ of 60.2 mmHg. On postoperative day 2, arterial blood gas analysis showed a pH of 7.470, a PaCO₂ of 25.3 mmHg, and a PaO₂ of 86.7 mmHg. On postoperative day 3, oxygenation gradually stabilized and corticosteroids and sivelestat sodium were discontinued. Postoperative analgesia consisted of intravenous opioid-based analgesia in the early postoperative period, followed by a combination of oral NSAIDs and opioids. No reintubation was required after extubation. From postoperative day 4 onward, arterial oxygenation returned to and remained within the normal range, and the patient was transferred out of the ICU after oxygen saturation had normalized.

**Fig. 3:**
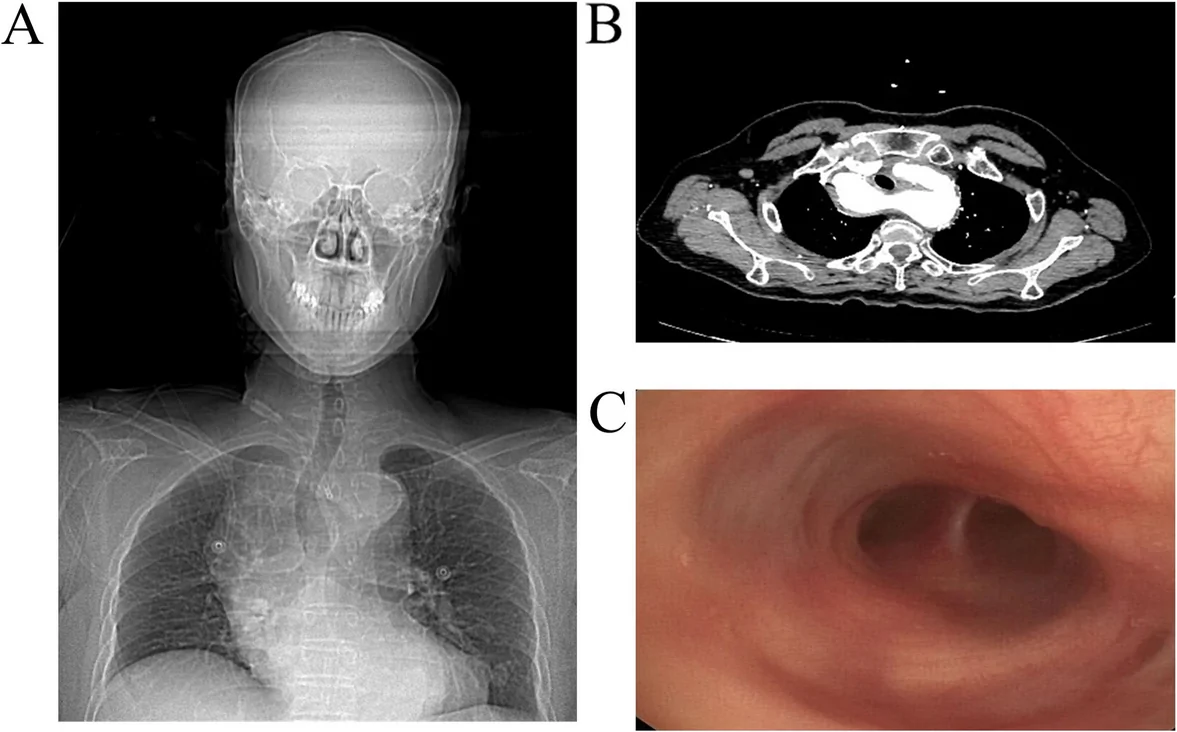
**A**–**C** Preoperative chest CT and postoperative bronchoscopy show no obvious tracheal stenosis or compression

**Fig. 4:**
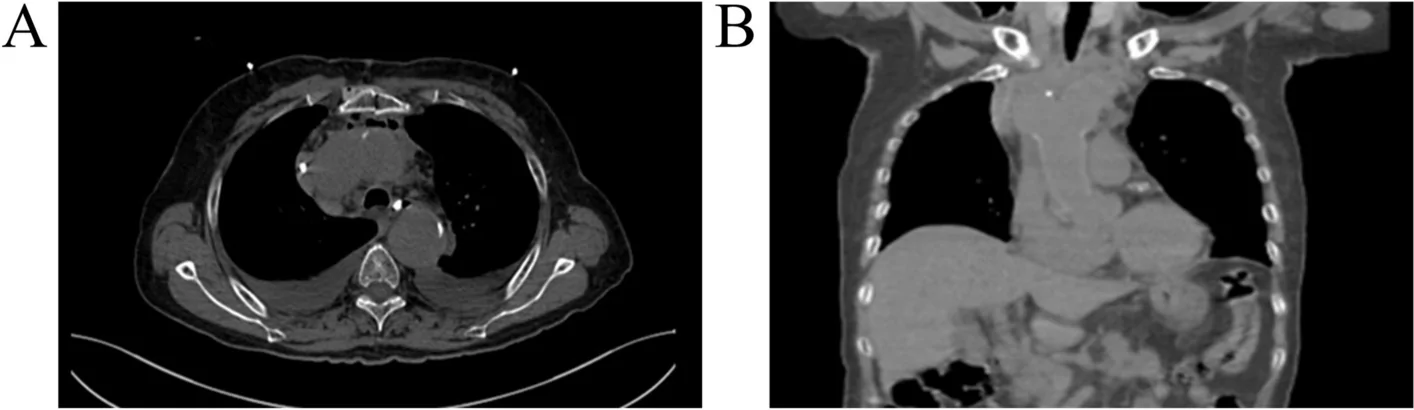
**A**, **B** Non-contrast chest CT on postoperative day 5 shows no remarkable abnormalities

Mediastinal and pericardial drainage decreased progressively after surgery, without massive bleeding or the need for re-exploration for hemostasis. Postoperative echocardiography showed a small amount of pericardial effusion without valvular regurgitation. Preoperative chest CT and postoperative bronchoscopy showed no obvious tracheal stenosis or compression (Fig. 3). Repeat chest CT on postoperative day 5 showed no remarkable abnormality (Fig. 4). Given the stable clinical condition and gradually decreasing drainage volume, the anterior mediastinal and pericardial drains were removed on the same day. The patient was discharged on postoperative day 12 and was instructed to maintain strict blood pressure control (systolic pressure 100–120 mmHg), continue regular oral antihypertensive therapy, such as a beta-blocker combined with an ACE inhibitor, and attend regular outpatient follow-up visits.

**Fig. 5:**
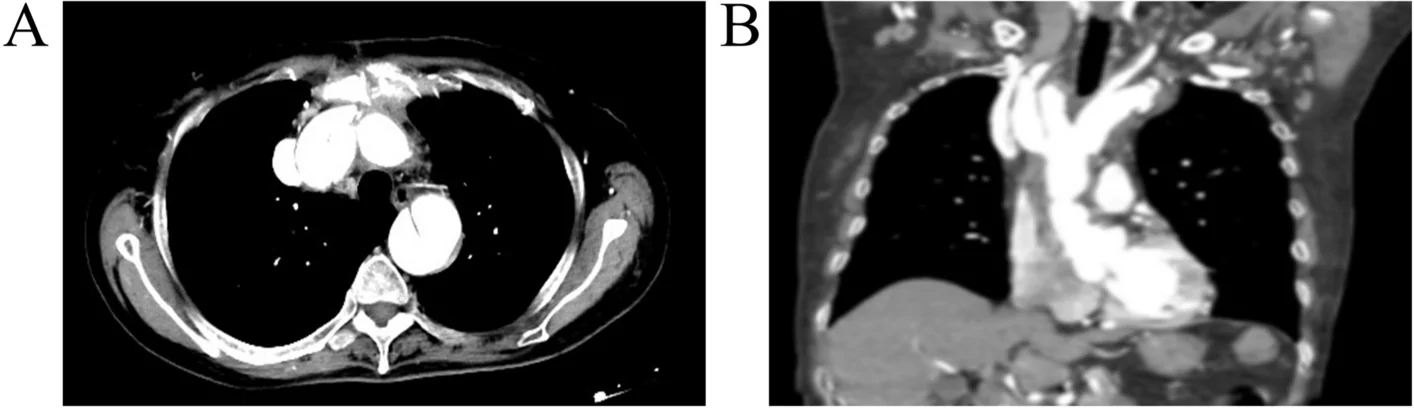
**A**, **B** CTA at 1 month postoperatively shows a newly developed dissection involving the proximal right aortic arch, without involvement of the anastomosis

Approximately 1 month after surgery, the patient presented again with severe chest pain. Blood pressure in the left upper limb on admission was 170/66 mmHg. Emergency whole-aorta CTA showed a newly developed dissection in the proximal right aortic arch, involving the unreplaced distal ascending aorta and arch and extending distally to the distal abdominal aorta. The intimal entry site was closer to the proximal right arch, without involvement of the anastomosis or the distal end of the graft (Fig. 5). Further history-taking revealed that the patient had not maintained regular blood pressure control after discharge. Reoperation was recommended; however, the family declined and chose conservative treatment at another hospital. The patient was subsequently lost to follow-up.

## Discussion

Double aortic arch results from abnormal embryologic regression of the aortic arches and accounts for less than 1% of congenital heart disease. Adult DAA is even rarer and is often asymptomatic or discovered incidentally [8]. DAA is frequently associated with other congenital cardiovascular malformations, and developmental abnormalities involving chromosome 22q11 are closely related to DAA [5, 9].

DAA complicated by AAS is exceedingly rare, and most reported cases have involved descending aortic disease or Kommerell diverticulum-related pathology; reports involving proximal aortic disease are scarce [10–13]. This case of adult DAA complicated by acute type A IMH with a focal intimal tear [14] suggests that the bounda ries of surgical strategy under complex anatomy require re-evaluation, as does the potential long-term price that may accompany a limited emergency approach.

The 2022 ACC/AHA guideline for aortic disease states that complicated IMH should undergo urgent repair and that type A IMH should also be repaired as early as possible [15]. In the present case, limited ascending aortic resection and graft replacement were performed under retrograde perfusion via right femoral artery cannulation, without simultaneous reconstruction of the two arches. The main reasons were as follows. First, the supra-aortic branches were involved and the branching anatomy of the double aortic arch was highly complex, making conventional axillary cannulation unreliable for cerebral perfusion. In addition, when the subclavian arteries are small and the local vessel wall is stiff and calcified, local pressure elevation may occur and could progress to dissection. Second, although the hematoma involved the right common carotid artery arising from the right arch, preoperative CTA suggested that the true lumen remained patent and there was no clinical evidence of cerebral ischemia. Third, the patient was elderly and frail, and extensive arch reconstruction with circulatory arrest was considered excessively risky in the emergency setting.

However, this limited surgical strategy should not be interpreted as a universally reproducible approach. In this case, it represented a compromise made for a frail elderly patient in the setting of complex DAA anatomy; for this patient, extensive arch reconstruction and deep hypothermic circulatory arrest were considered too hazardous. The major trade-off was that simultaneous clamping of both arches on potentially diseased and calcified vessel segments might have caused iatrogenic intimal injury, promoted progression of the IMH toward the supra-aortic branches, and even precipitated rupture or uncontrollable bleeding at clamp release [16]. In addition, without opening the arch, it was impossible to directly visualize the true distal extent of disease, thereby increasing the risk of insufficient resection margins and leaving behind a residual high-risk arch segment.

Tsukioka et al. [17] reported a case of DAA complicated by acute type A aortic dissection that was also managed with ascending aortic replacement by clamping both arches. Postoperative imaging showed residual DAA and residual dissection. Although that report did not describe a newly developed dissection at the clamping site, the persistent residual dissection still suggests that a conservative strategy of “ascending aortic replacement alone” has limitations and may fail to fully address distal disease or future complications. Toyokawa et al. [18] reported an elderly patient with DAA complicated by acute type A aortic dissection who underwent not only ascending aortic replacement but also partial hemiarch replacement, effectively addressing the arch lesion at the same operation. Together, these reports suggest that in DAA complicated by proximal acute aortic syndromes, the extent of surgery should be individualized, but more active arch management should be strongly considered when the anatomy and overall condition permit.

Regarding the persistent hypoxemia before surgery and in the early postoperative period, the patient had repeated preoperative hypoxemia and continued postoperative oxygenation impairment. Because preoperative CT did not show obvious infection, echocardiography showed no intracardiac shunt, and serial blood gas analyses did not indicate hypercapnia, pulmonary injury secondary to inflammatory mediators related to IMH should be considered the main explanation. Meanwhile, chronic airway compression caused by the DAA vascular ring may have been amplified during the perioperative period by airway mucosal edema, retained secretions, and changes in ventilation pressure. It should be noted that no preoperative airway three-dimensional CTA reconstruction or bronchoscopy was performed to quantify the degree of compression, and postoperative bronchoscopy also showed no obvious stenosis; therefore, the contribution of the vascular ring to hypoxemia lacks direct evidence. For similar patients, preoperative assessment of airway compression should be routinely considered, and when moderate-to-severe compression or recurrent respiratory complications are present, the benefits and risks of simultaneous or staged division of the vascular ring should be actively evaluated [19–22].

Although limited ascending aortic replacement in the emergency setting for DAA complicated by acute type A IMH with a focal intimal tear may reduce surgical invasiveness and short-term mortality, the postoperative risks and long-term prognosis should not be underestimated. In this case, under the background of poor blood pressure control or possible clamp-related injury, the residual arch hematoma progressed to a newly developed dissection. In future similar cases, dependence on arch clamping should be minimized whenever possible. If necessary, open distal anastomosis (ODA) under deep hypothermic circulatory arrest may be used so that the anastomosis can be created on a relatively normal segment under direct vision. When anatomy permits, more definitive simultaneous or staged management of the vascular ring and residual arch disease should also be considered [23, 24].

## Conclusions

In patients with double aortic arch complicated by type A intramural hematoma with a focal intimal tear, limited ascending aortic replacement in the emergency setting may reduce surgical trauma, but it may leave behind a high-risk residual arch segment and carries the potential risk of clamp-related injury and inadequate resection. Perfusion and arch-management strategies should be individualized on the basis of comprehensive three-dimensional assessment; when necessary, techniques such as ODA may reduce dependence on arch clamping. Perioperative assessment of airway compression, strict postoperative blood pressure control, and structured follow-up should be emphasized to achieve more durable aortic repair.

## Data Availability

No datasets were generated or analysed during the current study.

## Abbreviations

DAA: Double aortic arch
IMH: Intramural hematoma
PAU: Penetrating atherosclerotic ulcer
AD: Aortic dissection
AAS: Acute aortic syndrome
ICU: Intensive care unit
ACC/AHA: American College of Cardiology/American Heart Association
ATAAD: Acute type A aortic dissection
CPB: Cardiopulmonary bypass
TEVAR: Thoracic endovascular aortic repair
CT: Computed tomography
CTA: Computed tomography angiography
3D: Three-dimensional
ODA: Open distal anastomosis
